# “It's what we call a randomised control trial” exploring how randomisation is presented by recruiters in RCTs

**DOI:** 10.1186/1745-6215-16-S2-O36

**Published:** 2015-11-16

**Authors:** Marcus Jepson, Daisy Townsend, Julia Wade, Carmel Conefrey, Jenny Donovan

**Affiliations:** 1 University of Bristol, Bristol, UK

## Background

The way recruiters’ present information to patients can influence whether or not the patient agrees to participate in an RCT. We know that many clinicians find it difficult to communicate about trials, including the concept and justification of randomisation. Current clinical guidance states that patients must be informed about randomisation but there is little advice about how to do this in practice. The aim of this paper is to show how randomisation was presented by recruiters, and provide suggestions about key aspects that can inhibit/facilitate informed decision making.

## Methods

94 audio recordings of recruitment appointments across 5 RCTs were analysed. All references to randomisation were noted (including when it was not explained). Explanations of randomisation were then sorted into themes according to whether they related to the method or process of allocation, reason for randomisation or other elements.

## Findings

A small proportion of recruitment appointments did not include any reference to randomisation; in those that did, the method and process of allocation were mentioned regularly, but reasons for randomisation were rarely presented. Descriptions used by recruiters often drew on metaphors, sometimes with inaccuracy. Some explanations led to patients believing they would receive the treatment that was ‘best for them’.

## Conclusions

The method and process of randomisation are usually described by recruiters, but often awkwardly and with inaccuracy, but the rationale for randomisation is rarely mentioned. Recruiters require support and training to ensure patients are making fully informed decisions about RCT participation.

